# Conservative Management of a Rare Gastrobronchial Fistula Following Minimally Invasive Esophagectomy: A Case Report

**DOI:** 10.7759/cureus.99700

**Published:** 2025-12-20

**Authors:** Urmimala Chaudhuri, Evan Hartman, Jonathan R Forrest, Bahar Cheema, Mustafa Musleh

**Affiliations:** 1 Department of Internal Medicine, Wright State University, Dayton, USA; 2 Department of Internal Medicine, Wright State University Boonshoft School of Medicine, Dayton, USA; 3 Department of Gastroenterology, Wright State University, Dayton, USA; 4 Department of Gastroenterology, Premier Miami Valley Hospital, Dayton, USA

**Keywords:** anastomotic leakage, esophageal cancer, esophageal stent, esophagectomy, gastrobronchial fistula

## Abstract

A gastrobronchial fistula (GBF) is an uncommon condition in which an abnormal connection develops between the stomach and the lungs. It can occasionally arise as a complication following esophagectomy for esophageal adenocarcinoma. Management of GBF includes both surgical and nonsurgical approaches, depending on the patient’s condition and comorbidities. Conservative management focuses on diversion to promote fistula closure; options include esophageal stents, fasting, nasogastric drainage, proton pump inhibitors, and jejunostomy feeds. Surgical options include resection of the fistula, anastomosis revision, and closure of the gastric conduit and bronchial aperture. We present a rare case of a 78-year-old male diagnosed with a GBF nearly two years after a minimally invasive distal esophagectomy for esophageal adenocarcinoma. Multiple diagnostic modalities, including endoscopy, CT, and contrast esophagram, were used to establish the diagnosis. The patient elected stent placement from the esophagus to the distal stomach due to poor functional status and was maintained on jejunostomy tube feeds. He was discharged in stable condition. This case highlights a conservative approach to treating GBF using a self-expanding stent (Endo-Flex GmbH, Voerde, Germany) and X-Tack (Apollo Endosurgery, Inc., Austin, TX, USA), an endoscopic suture device used to anchor the stent.

## Introduction

A gastrobronchial fistula (GBF) is an abnormal connection between the stomach (often a gastric conduit) and the lungs (pleural space) [1]. It is a rare complication that can occur following esophagectomy for esophageal cancer, with an estimated incidence of 0.3-5.6% [1]. Previous studies have shown that GBFs are typically diagnosed at a median of two months after esophagectomy [2]. GBF is associated with high mortality, and its clinical presentation can be variable, including fever, cough, dysphagia, chest pain, dyspnea, pneumonia, and hemoptysis [1-4]. Therefore, maintaining a high index of suspicion in cases of sudden clinical change is important for prompt diagnosis [3]. Early recognition and management can prevent disease progression and improve quality of life [1].

Multiple imaging modalities are used to establish the diagnosis, including endoscopy, contrast esophagogram, CT, and bronchoscopy [3]. Currently, there is no widely accepted management strategy for GBF, likely due to the extreme rarity of the condition and the heterogeneity of causes and presentations. Management options include surgical repair, stent placement, and conservative approaches [1-3].

We present a rare case of a patient diagnosed with a GBF after a minimally invasive distal esophagectomy for esophageal adenocarcinoma. We discuss the presentation of GBF and outline an approach to conservative management.

This article was previously presented as a meeting abstract at the 2024 American College of Gastroenterology (ACG) Annual Meeting on October 27, 2024.

## Case presentation

A 78-year-old male with a history of smoking initially presented with an esophageal nodule detected during endoscopy, which was suspicious for malignancy. Initial CT of the abdomen and pelvis with contrast showed mild thickening of the mid-esophagus without other acute findings suggestive of metastatic disease. Pathology confirmed well-differentiated adenocarcinoma of the esophagus. PET demonstrated focal increased uptake corresponding to the known malignancy.

The patient underwent minimally invasive laparoscopic esophagogastrectomy with intrathoracic anastomosis, Botox pyloroplasty, and placement of a feeding jejunostomy tube (J-tube). Final pathology revealed a 4-cm well-differentiated adenocarcinoma invading the muscularis propria, with negative margins and five of 15 lymph nodes positive (Stage IIIB). Neoadjuvant chemoradiation was not administered, as preoperative imaging and endoscopic evaluation suggested early-stage disease. No anastomotic leak was identified, and the patient was discharged on postoperative day 7.

One month after surgery, the patient returned to the emergency department and was admitted for septic shock and right-sided empyema. During hospitalization, esophagogastroduodenoscopy (EGD) revealed necrotic gastric mucosa below the gastroesophageal anastomosis, involving the entire circumference. A large leak distal to the anastomosis was identified, and a covered Wallstent was placed in the esophagus. Right pleural fluid cultures grew *Enterobacter*, *Enterococcus*, and *Streptococcus constellatus*. Later during this admission, approximately one month after these interventions, the patient underwent full-mouth dental extraction due to concern for oral bacterial contamination contributing to persistent lung infection. He was discharged on strict nil per os (NPO) status with J-tube feeds. The esophageal stent was removed three months later, as a repeat CT scan with contrast showed no evidence of an anastomotic leak.

**Figure 1:**
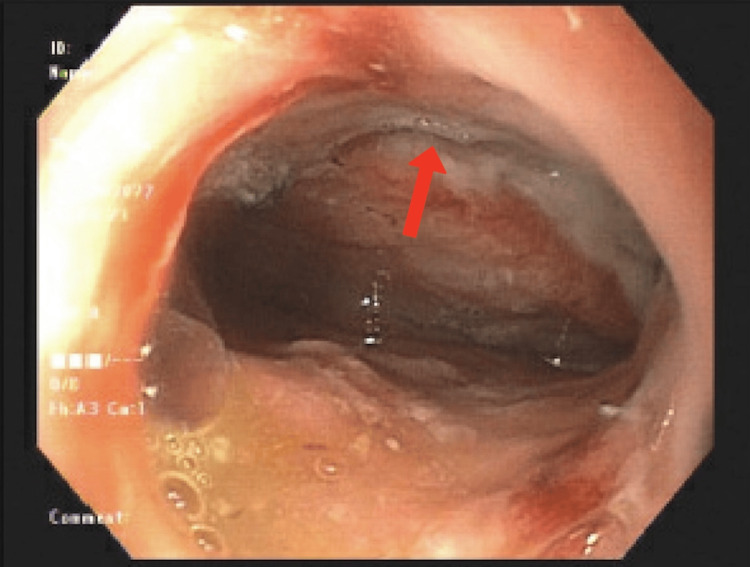
EGD showing a large patch of gastric ulceration with complete loss of mucosa distal to the anastomosis (red arrow) EGD: esophagogastroduodenoscopy

**Figure 2:**
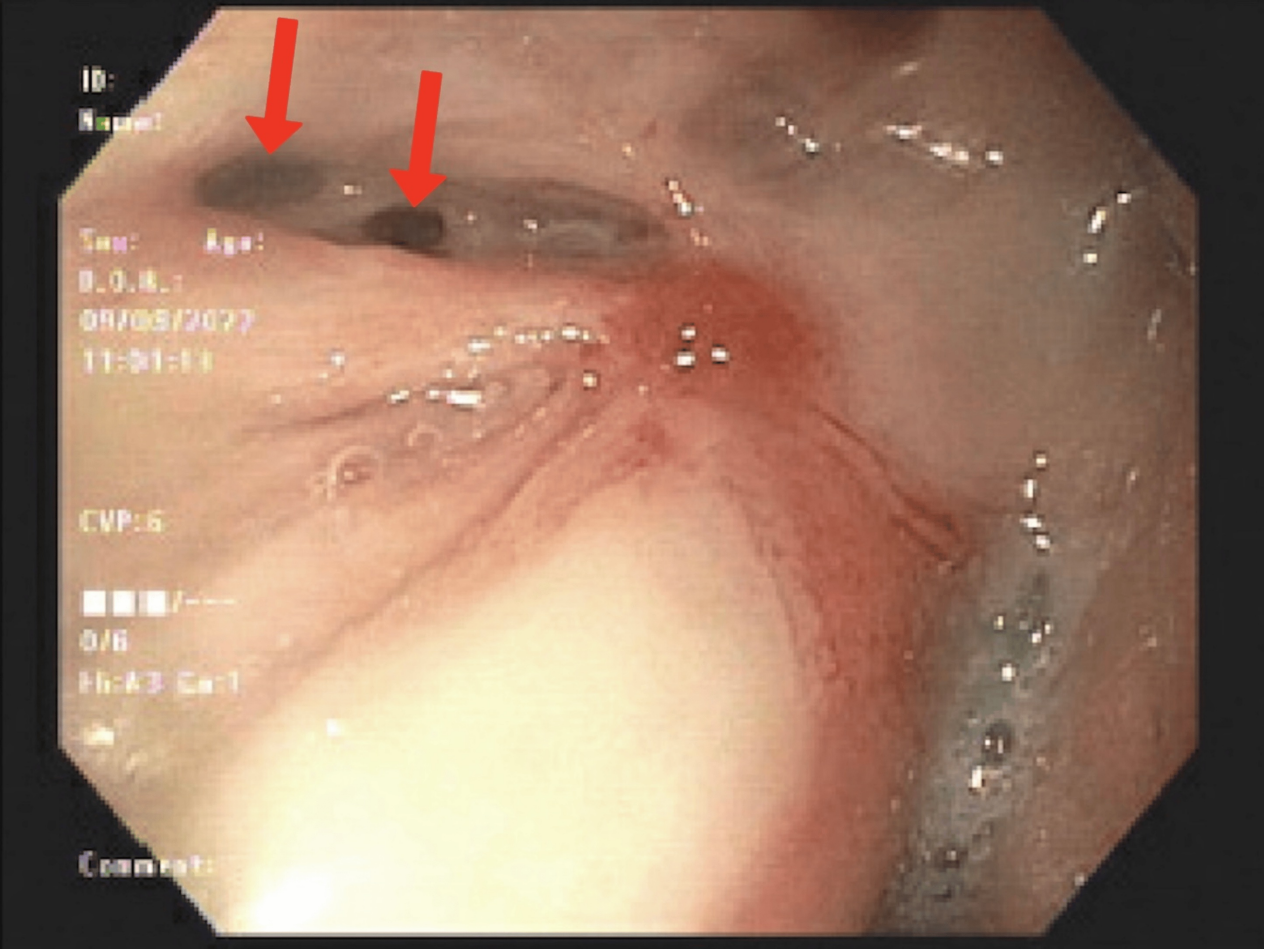
EGD showing two 1-2 mm perforations or fistula openings (red arrows) EGD: esophagogastroduodenoscopy

Due to poor oral intake, the patient underwent a barium esophagram, which revealed retention of food proximal to the anastomosis. He had also experienced several weeks of shortness of breath and abdominal bloating. Based on the esophagram findings, EGD was performed for dilation of the esophageal anastomotic stricture and Botox injection to the pylorus. EGD also revealed a large patch of gastric ulceration with complete loss of mucosa distal to the anastomosis (Figure 1) and two 1-2 mm perforations or fistula openings (Figure 2).

**Figure 3:**
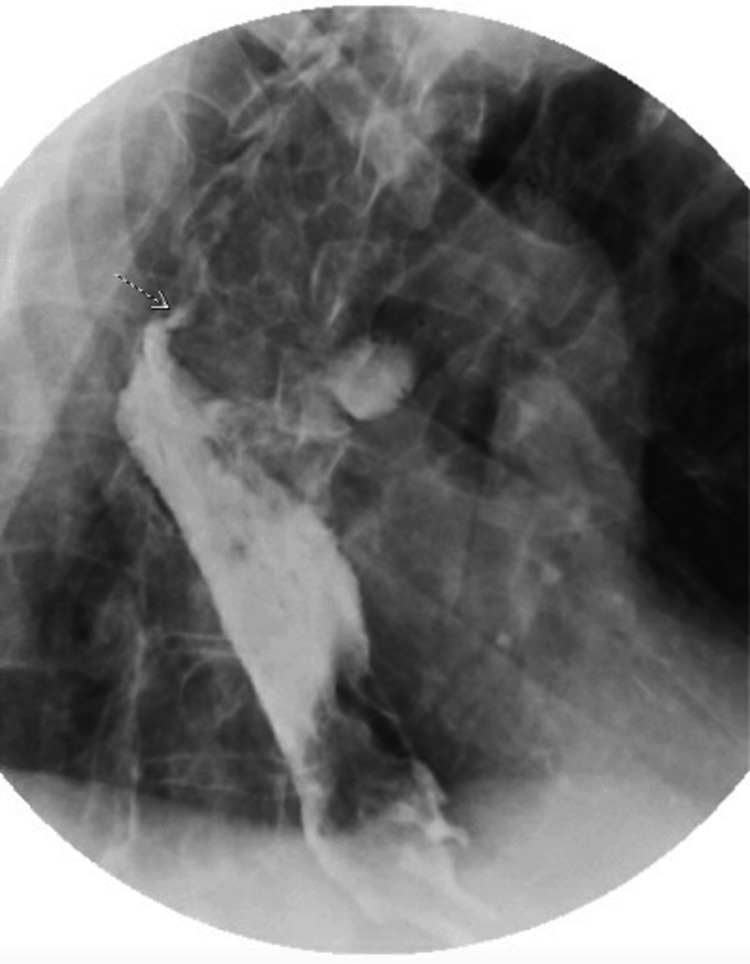
Esophagram showing extravasation from the stomach into bronchial structures, specifically the right upper lobe of the lung (dotted arrow)

**Figure 4:**
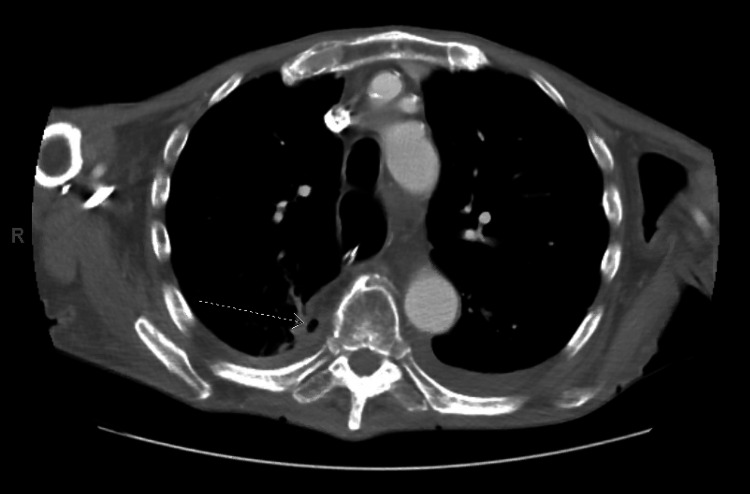
Axial CT of the chest with contrast showing a GBF (dotted arrow) GBF, gastrobronchial fistula

An esophagram demonstrated slow extravasation from the stomach into bronchial structures, specifically the right upper lobe (Figure 3), strongly suggestive of a GBF. CT of the chest with contrast confirmed a GBF connecting the superior margin of the gastric pull-through at the anastomotic site to the posterior medial pleural space (Figure 4). Bronchoscopic confirmation was not pursued due to the patient’s poor functional status, as the imaging findings were sufficient to establish the diagnosis of GBF.

The patient was considered a poor candidate for open fistula repair due to poor functional status, as evidenced by malnutrition related to stent placement, frequent hospital readmissions, overall weakness, and inability to perform activities of daily living independently. He elected for stent placement from the esophagus to the distal stomach. During the procedure, a self-expanding 23 mm × 12 cm Endo-Flex stent (Endo-Flex GmbH, Voerde, Germany) was deployed in the esophagus and secured with X-Tack (Apollo Endosurgery, Inc., Austin, TX, USA). The X-Tack was anchored 1 cm proximal to the stent and at the upper edge of the stent to minimize the risk of stent migration. The patient was discharged in stable condition with a strict NPO status and jejunostomy tube feeds until follow-up evaluation.

## Discussion

This case highlights gastrobronchial fistulization, a rare but potentially life-threatening complication of esophagectomy. GBF arises from the abnormal connection between the airway and the stomach, typically resulting from upward mobilization and anastomosis of the gastric conduit to the remaining esophagus [1]. Risk factors include prior gastric or esophageal surgery, abscesses, trauma, ulcers, and neoplasms [5]. Our patient underwent a distal esophagectomy for esophageal adenocarcinoma.

Early postoperative complications following esophagectomy include pleural effusion and gastric stasis, while the most frequent late complication is anastomotic stricture. The reported frequency of GBF after esophagectomy ranges from 0.04% to 3% [3,4], with other studies estimating 0.3-5.6% [6]. Anastomotic leaks account for approximately 40% of post-esophagectomy deaths [7]. Ischemia in the walls surrounding the staple line is considered the primary cause of leaks and subsequent fistulas [8].

Patients who develop a GBF may present with symptoms ranging from a mild cough to severe bronchopneumonia or mediastinitis. Other reported symptoms include chest pain, dyspnea, hemoptysis, fever, and expectoration of gastric contents. Esophagogram has a sensitivity of 78% for detecting GBF [7], while CT imaging may reach 92% sensitivity when fistulas are suspected [9]. Endoscopy and bronchoscopy are also utilized for diagnosis.

Treatment strategies depend on the location and size of the fistula, the patient’s overall condition and comorbidities, and whether the GBF occurred early or late postoperatively. Early causes of GBF include esophageal anastomotic leaks, while late causes may include nonhealing gastric conduit ulcers, anastomotic leaks, tumor recurrence, or radiation injury [10]. In the absence of sepsis, conservative management is a reasonable approach [11]. Conservative measures include fasting, nasogastric drainage, proton pump inhibitors, and jejunostomy feeds. However, if the fistula fails to heal within one to two months, escalation beyond conservative management is recommended [12].

Other nonsurgical approaches include esophageal stents, such as self-expanding stents, over-the-scope clips, or silicon stents. Endoscopic procedures with drainage suggest that stents, clips, and tissue adhesives may be successful in 80-95% of cases [9]. Surgical management of aerodigestive fistulas (ADFs) shows postoperative mortality of 2.8% and morbidity of 12-54% for benign ADFs, compared to 14% mortality and 40% morbidity for malignant ADFs [13]. Recurrence rates have been reported at 26% for airway stents, 16% for esophageal stents, and 10% for double stents [13], with other studies reporting overall recurrence ranging from 13% to 66% [13,14]. Successful fistula occlusion is associated with a mean survival of 242 days, compared to 80 days in patients with stent failure [13]. Airway stenting has been shown to improve survival compared to supportive management (69 vs. 29 days and 120 vs. 55 days in separate studies) [13]. Stent migration is a common complication, reported in 15-60% of cases [9].

The choice of treatment is influenced by the fistula’s configuration and location, as well as the patient’s overall condition and comorbidities. It is critical for patients with GBF to remain NPO to prevent aspiration of gastric contents; in our case, the patient received strict jejunostomy tube feeds.

If nonsurgical approaches fail, surgical management may be required. Surgical options include anastomosis revision, resection of the fistula, and closure of the gastric conduit and bronchial aperture. These procedures are typically reserved for benign GBF cases. While surgical repair is considered definitive, outcomes remain variable due to limited data [10,15]. Surgical interventions may be more reliable than nonsurgical approaches, but the rarity of these fistulas limits the available evidence. Given the heterogeneity and low incidence of GBF, no standardized treatment algorithm or surgical guidelines exist, highlighting the need for further research in this area.

## Conclusions

GBF is a rare complication of esophagectomy for esophageal adenocarcinoma. This case highlights the option of nonoperative, conservative management for patients who are not optimal surgical candidates. It also emphasizes the importance of considering GBF in patients presenting with an anastomotic leak or gastrointestinal and/or respiratory symptoms following esophagectomy. Early diagnosis and multidisciplinary management are critical, as they can help prevent disease progression and improve quality of life.
